# Clinical assessment and treatment of patients presenting with longstanding hip and groin pain in primary care: a survey study among physical therapists and general practitioners in Sweden

**DOI:** 10.1186/s12891-025-08466-6

**Published:** 2025-03-03

**Authors:** August Estberger, Kristian Thorborg, Harald Talts, Eva Ageberg

**Affiliations:** 1 Department of Health Sciences, Faculty of Medicine, Lund University, Lund, Sweden; 2 Sports Orthopedic Research Center– Copenhagen (SORC-C), Department of Orthopedic Surgery, Amager-Hvidovre Hospital, Institute for Clinical Medicine, Copenhagen University, Copenhagen, Denmark

**Keywords:** Hip pain, Groin pain, Primary care

## Abstract

**Background:**

Methods of assessment, treatment and referral rates of patients presenting with longstanding hip and groin pain (LHGP) are not well documented. The aim of this study was to investigate assessment and treatment of patients with LHGP among general practitioners (GPs) and physical therapists (PTs) in primary care.

**Methods:**

An anonymous web-based survey was developed specifically for this study and distributed to GPs and PTs at primary care centers in the southern part of Sweden. The survey covered the use of different methods of assessment and treatment for LHGP, the perceived importance these methods, and referral rates to orthopedic care. Responses from clinicians were reported in frequencies and percentages, and differences in assessment methodology between professions were examined with chi-square tests.

**Results:**

PTs (*n* = 104) and GPs (*n* = 62) referred less than 25% of patients with LHGP to orthopedic care. Both professions used clinical assessments as range of motion tests, but PTs were more likely to use specific clinical tests (PTs 76% vs. GPs 19%, p = < 0.001), GPs used more imaging (GPs 98% vs. PTs 58%, p = < 0.001) and neither profession used validated patient-reported outcome measures (GPs 2% vs. PTs 11%, *p* = 0.134). GPs and PTs ranked patient history and range of motion as the most important factors for diagnosis. GPs and PTs both reported providing patient education and advice on physical activity as part of the treatment. GPs commonly prescribed pain medication, including NSAIDs (97%), paracetamol (100%), and opioids (69%). 77% of PTs reported treatment duration less than 3 months, with treatment consisting of combinations of exercise therapy and manual therapy.

**Conclusions:**

GPs and PTs in primary care referred 25% or less of patients with LHGP to orthopedic care. Both professions generally used assessment for LHGP in line with clinical recommendations. However, some assessment methods differed between GPs and PTs, and neither used validated patient-reported outcome measures. Treatment strategies mainly included pain medication (GPs), exercise and manual therapy (PTs), and education (both professions). Inconsistent with clinical recommendations, GPs commonly prescribed opioids as part of pain management, and PTs report treatment duration of less than 3 months.

**Clinical trial number:**

NA.

**Supplementary Information:**

The online version contains supplementary material available at 10.1186/s12891-025-08466-6.

## Background

Non-arthritic longstanding hip and groin pain (LHGP) can arise from intra- and/or extra-articular structures [1], such as femoroacetabular impingement and adductor tendinopathy. Diagnosing the structural contributors to LHGP presents a challenge [2], due to overlapping presentations [1], and low specificity of clinical tests and imaging [3]. In a cohort study, about 50% of patients referred to an orthopedic department at a university hospital were classified as having intra-articular pain [1], suggesting that a large proportion of patients referred are not eligible for hip arthroscopy. Improved diagnosis and management of patients presenting with LHGP in primary care may lower societal costs and reduce delayed care for patients in need of surgery.

Primary care is often the first line of assessment and treatment of musculoskeletal disorders. In Sweden, the primary care system consists of primary health care centers, where general practitioners (GPs), nurses and physical therapists (PTs) provide basic medical assessment and treatment [4]. GPs and PTs in Sweden act as autonomous clinicians, and PTs are often the first contact practitioner for musculoskeletal complaints [5]. GPs and PTs may also refer patients to orthopedic care for further investigations. This is similar to many of the medical systems internationally, as direct access to physical therapists in some capacity has been reported in 58% of countries [6], with similar health-related outcomes and patient satisfaction compared to primary management by GPs [7].

While most patients presenting with LHGP will go through assessment and first line treatment in primary care, most research on LHGP has been conducted in orthopedic settings or in organized sports, as summarized in systematic reviews [3, 8]. Cross-sectional and retrospective studies have reported that patients presenting with LHGP have often seen several treatment providers [1, 9, 10], and about 50% of patients referred to an orthopedic department had not received a diagnosis in primary care [11].

Some guidelines for the assessment and treatment of patients presenting with LHGP exist [3, 12–15]. The Doha agreement on classification of groin pain in athletes [15], the Warwick agreement on femoroacetabular impingement syndrome (FAIS) [12], and the Zurich consensus on hip-related pain are all consensus statements [3, 13], attempting to create unified terminology in LHGP. Clinical assessment of LHGP is recommended to include patient history and symptoms, clinical tests, hip and groin specific patient-reported outcome measures (PROMs) and, if indicated, imaging [3, 12, 14]. Clinical practice guidelines for most musculoskeletal disorders [16], including LHGP [12–14], recommend activity modification, advice, exercise therapy and medication as the primary intervention.

Clinical experts in groin pain have adopted the terminology and diagnostic criteria from the Doha agreement [17]. However, it is unknown if these developments in the field of hip and groin pain have been disseminated to general practice, as there are no studies describing current clinical practice for LHGP among GPs and PTs in primary care.

The aim of this study was to investigate GPs and PTs in primary care management of people presenting with LHGP, including assessment, treatment and referral rates to orthopedic care. In addition, we aimed to compare assessments used by GPs and PTs for diagnosing LHGP.

## Methods

### Study design

Cross-sectional survey study adhering to the Checklist for Reporting of Survey Studies (CROSS) guidelines [18].

### Data collection

The authors (3 physical therapist researchers, whereof 2 also clinically active, and 1 medical student) developed a study specific survey. The authors have experience assessing and treating patients with LHGP in orthopedic and primary care settings, as well as experience in conducting research in the musculoskeletal field. The final 27-item survey contained 4 questions regarding respondent demographics, 9 questions regarding assessment and 14 questions regarding treatment of patients with LHGP (see additional file 2 for survey questions, in Swedish (original version) and translated to English). Questions were designed to identify the use of different methods of assessment and treatment, as well as ask respondents to rank these methods by perceived importance. Twenty-four questions were multiple choice (of which 5 allowed respondents to add optional free text information), and 3 were ranking questions. The authors did not include strict definitions of tests and treatment methods to avoid influencing respondents by providing too much information. Thus, specific terms were left for the individual clinician to interpret.

The survey was distributed through SUNET Survey (SUNET, Stockholm, Sweden), a web-based survey system available through Lund University. The study setting was primary care centers in Skåne and Blekinge (southern Sweden). It was initially open for approximately three weeks, between late October and late November 2022. Due to a low response rate among GPs, the survey was re-opened for GPs during February 2023.

The introductory paragraphs of the survey presented respondents with a clinical scenario to consider while completing the survey. They were asked to consider a patient with: (1) Pain primarily from the groin area for more than 3 months; (2) Clinical suspicion of pain arising from the hip joint or local extra-articular structures; (3) Pain provoked by load and/or positioning of the hip/groin; (4) With or without one of the following patient histories: feeling of stiffness, clicking/catching from the groin area and/or a feeling of giving way, and (5) without red flags (infections, tumours or necrosis) or verified hip osteoarthritis.

### Respondents

Regional governing bodies provided contact information to operational managers of public and private health care centers in Skåne and Blekinge in Sweden. One researcher (HT) contacted these operational managers (*n* = 212) and asked them to forward the survey to GPs and PTs at their respective clinic. The authors also sent the operational managers a reminder email after one and two weeks. Ten email addresses were faulty and 3 operational managers declined the request to forward the survey. As the survey was sent to operational managers rather than the individual clinicians, it is unknown how many clinicians received the survey link. Based on records from the regional governing bodies, a total of 743 clinicians were active in the geographical area (GPs *n* = 410, PTs *n* = 333). In order to recruit respondents that assess and treat patients presenting with LHGP somewhat frequently, the inclusion criteria were GPs and PTs who had at least 1 year of clinical experience and treated at least 2 patients with LHGP per year. As it is not expected that all GPs and PTs fulfill these inclusion criteria, due to various clinical specialization, the target population is anticipated to be smaller than the total estimates of clinicians provided by the regional governing bodies. The exact target population size is unknown, as the respondents judged their own eligibility before choosing whether to respond to the survey.

### Statistics

No sample size calculation was performed due to the descriptive nature of the study. As the size of the target population (i.e. GPs and PTs with > 1 year clinical experience, treating > 2 patients with LHGP per year) is unknown, it is a challenge to estimate what constitutes a representative sample. As such, the authors attempted to reach as large amount of the total population as possible by sending the survey to all known primary care clinics. Also, a target was set of > 50 respondents from each profession.

All data analysis was performed in SPSS 29 (IBM Corporation, Armonk, NY). Descriptive data was summarized with frequencies and percentages. Chi-square tests with alpha set at 95% were used to analyze between-group differences in assessment methodology. Due to differences in professional role and scope of practice, the authors did not analyze differences in treatment strategies between GPs and PTs. For ranking questions, weighted average was calculated and used to determine rank for each profession. Some questions also had a free text option, but as the responses did not contribute to the aim of the study, they were not presented or analyzed.

### Ethical considerations

As per the Swedish law on ethics of research on human subjects [19], a survey only needs ethical approval from the Swedish Ethical Review Authority if it handles personal data and/or sensitive information, such as health status or political views [20]. As the current survey did not contain any sensitive information and was anonymous, no ethical approval was needed. Informed consent was not collected due to the above legislation, however, all participants were informed of the purpose of the study and that participation was voluntary. During data collection, responses were collected on the SUNET survey tool, a password protected service, and after completion of data collection, all data was downloaded and stored on a secure server at Lund University.

## Results

### Survey distribution and responder demographics

Thirty-five GPs, and 102 PTs responded to the initial survey. The second data collection generated an additional 31 responses (GP *n* = 27, PT *n* = 4). Two PTs that responded to the survey were excluded from the analysis due to reporting treating < 2 patients with LHGP per year. In total, 62 GPs and 104 PTs were finally included (Table 1). This represents 15% and 31% of GPs and PTs estimated to be clinically active in Skåne and Blekinge (GPs *n* = 410, PTs *n* = 333), however no response rate could be calculated for the target population, i.e., those assessing and treating patients with LHGP, as the size of this population is unknown.

**Table 1:** Respondent demographics

	GPs (*n* = 62) [n (%)]	PTs (*n* = 106) [n (%)]
**Working sector**		
Public	41 (66)	83 (78)
Private	15 (24)	18 (17)
Private and public	6 (10)	5 (5)
**Clinical experience**		
<2 years	-	14 (13)
2–5 years	14 (23)	21 (20)
6–10 years	14 (23)	19 (18)
11–20 years	13 (21)	20 (19)
>20 years	21 (34)	32 (30)
**Frequency of treatment of patients with LHGP**		
Every week	11 (18)	59 (56)
A couple times a month	33 (53)	35 (33)
A couple times bi-annually	18 (29)	9 (8)
A couple times annually	-	1 (1)
One time per year or more seldom	-	2 (2) *

LHGP = longstanding hip and groin pain. GPs = General practitioners. PT = Physical therapists. *n = 2 excluded due to not meeting inclusion criteria

### Referral rates

The majority of GPs (54%) and PTs (75%) referred less than 25% of their patients to orthopedic care.

### Assessment

GPs and PTs used a combination of diagnostic and impairment-based tests (Table 2). Patient history was ranked as the most important in making a diagnosis, with both professions rating it as extremely important (GPs 68%, PTs 85%). GPs ranked isometric pain provocation and PTs ranked imaging as the least important test. See Additional file 1 for details on ranking and perceived importance.

PTs used assessments of impairments, as well as specific tests and isometric tests to a greater degree than GPs, while GPs used imaging to a greater extent (Table 2). Few respondents reported using dynamometry (GPs 3%, PTs 4%) or hip-specific PROMs (GPs 3%, PTs 11%). Both GPs and PTs ranked tests of physical function, such as single leg squats, as the most important and dynamometry as the least important test for assessing impairments (Additional file 1).

**Table 2:** Survey responses regarding assessment

	GPs (*n* = 62) [n (%)]	PTs (*n* = 104) [n (%)]	*p* value
**Assessment with diagnostic tests**			
Hip ROM	62 (100)	104 (100)	1.0
Palpation	61 (98)	96 (92)	0.094
Isometric tests for pain provocation	34 (55)	100 (96)	< 0.001
Specific tests (e.g., FADIR, FABER)	12 (19)	79 (76)	< 0.001
Imaging methods	61 (98)	60 (58)	< 0.001
**Assessment of impairments**			
Tests of function	39 (63)	102 (98)	< 0.001
Manual muscle strength tests	35 (57)	98 (94)	< 0.001
Muscle tightness	25 (40)	87 (84)	< 0.001
Translatory movement of the hip	12 (24)	39 (38)	0.010
Isometric dynamometry (strength)	2 (3)	4 (4)	0.836
Pain scales (e.g., VAS, NRS)	34 (55)	92 (89)	< 0.001
PROMs for hip function	2 (3)	11 (11)	0.134

GPs = general practitioners, PTs = physical therapists, ROM = range of motion, FADIR = Flexion, ADduction, Internal Rotation test, FABER = Flexion ABduction External Rotation test, VAS = visual analog scale, NRS = numerical rating scale, PROMS = patient reported outcome measures

### Treatment

GPs and PTs used a combination of medication, advice and exercise therapy as treatment for LHGP (Table 3). GPs (69–100%) commonly prescribed analgesics (including NSAIDs, paracetamol and opioids), and PTs provided a combination of exercised-based interventions (100%) as well as manual treatment (68%). Both professions reported using patient education strategies (69–99%). GPs ranked physical activity as the most important for adjunct treatment, PTs ranked exercise therapy the highest, while both professions ranked passive treatments as the least important (Additional file 1). 77% of PTs reported intervention duration shorter than 3 months.

**Table 3:** Survey responses regarding treatment

	GPs (*n* = 62) [n (%)]	PTs (*n* = 104) [n (%)]
**Recommendations of analgesics**		
Paracetamol	62 (100)	62 (60)
NSAID (pill)	60 (97)	58 (56)
Opioids	43 (69)	-
NSAID (local)	22 (36)	17 (16)
Tramadol	13 (21)	-
**Recommendations of exercise**	60 (97)	106 (100)
ROM exercises	51 (94)	101 (98)
Strength exercises	45 (83)	103 (100)
Stability exercises/ neuromuscular exercises	32 (59)	91 (88)
Plyometric exercises	5 (9)	26 (25)
**Recommendations of physical activity**	61 (98)	104 (100)
Everyday exercise	58 (97)	102 (98)
Endurance exercise	52 (87)	102 (98)
Sports	12 (20)	36 (35)
**Recommendations of passive treatments**	28 (45)	71 (68)
Acupuncture/TENS	25 (40)	49 (49)
Joint mobilisation	5 (14)	44 (44)
Tape/belts	7 (20)	36 (36)
Soft tissue mobilisation	6 (17)	26 (26)
**Information to patients with LHGP**		
Pain/worries	59 (95)	103 (99)
Treatment alternatives	59 (95)	100 (96)
Prognosis	55 (89)	99 (95)
Explanation of anatomical structures	43 (69)	102 (98)
Pathophysiological aetiologies	42 (68)	87 (84)

GPs = general practitioners, PTs = Physical therapists, NSAIDs = Non-steriodal anti-inflammatory drugs, ROM = range of motion, TENS = transcutaneous electric nerve stimulation, LHGP = longstanding hip and groin pain

## Discussion

In the current study, management of patients presenting with LHGP among GPs and PTs in primary care was surveyed. The majority of both GPs (54%) and PTs (75%) referred less than 25% of their LHGP patients to orthopedic care. GPs and PTs reported using patient history and hip ROM as important parts of the diagnostic assessment. GPs used imaging to a greater extent than PTs, while PTs used isometric pain provocation and specific tests more than GPs. PTs were more likely than GPs to assess impairments using tests of function, manual muscle strength tests, muscle tightness testing, translatory movement of the hip joint and pain scales. Neither profession used dynamometry to assess hip muscle strength or hip-specific PROMs for evaluation of patient-reported hip-related quality of life.

In treatment both professions reported using patient education, including addressing concerns about pain, available treatment options, prognosis and pathophysiological explanations. GPs used a variety of anesthetics, including paracetamol, NSAIDs and opioids. PTs reported treated LHGP using a combination of exercise therapy and manual therapy, with treatment duration usually lasting less than 3 months.

Guidance on the assessment and treatment of hip-related pain and FAIS exists as clinical practice guidelines and consensus statements [3, 12–14]. It is recommended that the diagnostic procedure includes a combination of patient history, specific tests, and imaging, as well as the use of hip-specific PROMs [3, 12, 14]. In the current study, PTs were more likely to use specific tests, and GPs were more likely to use radiographic imaging, while neither profession used hip-specific PROMs or dynamometry. Assessment of hip muscle strength is considered an important part of assessment of impairments in LHGP [21], and an association has been reported between improvements in adductor force production and better PROMs scores [22, 23]. Hand-held dynamometry is reliable for assessing hip strength [24], and is a more reliable and sensitive measurement tool than manual muscle tests [25]. In the current study, both GPs and PTs ranked manual muscle strength tests the second most important test for assessing impairments while dynamometry was ranked the least important. This could indicate a lack of knowledge of the measurement properties of the different methods and/or a lack of availability of hand-held dynamometers in primary care. It appears that both GPs and PTs fail to fully follow current recommended clinical practice, as some of the recommended assessment tools are not used. As neither PROMs or dynamometry was used, it is unclear how patient response to treatment is monitored, or how care progression is motivated.

In the current study, both GPs and PTs used advice and patient education as part of treatment, in line with current clinical recommendations [14, 16], but also somewhat different treatment strategies. GPs commonly prescribed analgesics. Even though restrictions and limited use of opioids for musculoskeletal pain is recommended [26], 69% of GPs reported prescribing opioids as part of pain management. This indicates that people with LHGP in primary care are subjected to drug prescription not supported by evidence. PTs used a combination of exercise therapy and manual techniques as part of management. The vast majority (89–100%) of PTs reported using ROM, strength and stability exercises as part of exercise therapy. The optimal type and dosage of exercise therapy for LHGP has not been determined, but this multimodal approach largely falls in line with recommended practice [13, 14]. However, most PTs reported treatment duration less than 3 months, while clinical recommendations has been suggested to last at least 3 months [13]. Thus, physical therapist-led treatment in primary care may not be of sufficient duration to elicit the optimal treatment response.

In the current study, GPs and PTs differed in assessment and treatment of LHGP, which is in line with finding from studies on other musculoskeletal conditions, such on low back pain [27] and hip disease [28]. Even in areas with more clearly defined clinical guidelines, clinicians do not always adherence to these in clinical practice [29]. Potentially, professional roles and bias can influence the perceived value of assessment tools and treatment strategies.

Results from the current study indicates that most patients presenting with LHGP do not get referred to orthopedic care. As the majority of participants in research studies to date have been recruited from orthopedic settings [3, 30], this could denote a potential misrepresentation of people with LHGP in the published literature. More research on people with LHGP in primary care is needed to enable a better understanding of the trajectory and identification of LHGP. For example, a pilot RCT recruiting patients with FAIS from advertisements to the general population reported significantly better iHOT-33 scores [23], compared to those included in studies conducted in orthopedic settings [31]. This could imply a different patient population, highlighting the potential benefit of implementing early best practice assessment and interventions in primary care.

### Strengths and limitations

To the authors knowledge, this is the first study to investigate assessment and treatment of patients with LHGP among GPs and PTs in primary care. The results can help improve clinical practice in primary care by identifying areas in need of implementation of best practice guidelines, such as opioid prescription.

The main limitation is that the total eligible sample is unknown. As the survey was distributed to clinic managers, it is unknown how many of the estimated sample population received the survey. Also, it is unknown how many potential respondents considered themselves ineligible due to the inclusion criteria of seeing at least 2 patients with LHGP per year. Therefore, the response rate should be interpreted with caution. Also, due to the descriptive nature of the study, no sample size calculation was performed. There is a potential for a non-representative sample as clinicians who are more knowledgeable and/or interested in LHGP may be more likely to respond to the survey. For these reasons, the survey responses may not be completely representative of the target population. However, this risk should be somewhat mitigated by the broad distribution of the survey to all known primary care clinics in the region.

The authors did not provide definitions of all variables in the survey, so the interpretation of the survey questions was to some extent up to the individual clinician. This was done in an effort to avoid influencing the respondents, but may have led to different interpretations of the various terms used in the survey. For example, the knowledge in strength exercise prescription has been reported to vary between physical therapists [32], so what constitutes strengthening exercises may differ between respondents. As such, specific conclusions about treatment strategies used in primary care cannot be drawn from the current study. Also, as the survey was developed specifically for this study, the questionnaire has not been validated. However, the responses in the survey still provide an overall picture of current clinical practice for assessment and treatment of patients with LHGP among GPs and PTs in primary care in Sweden.

Lastly, the specific effect of age, comorbidities or specific diagnosis were not considered in the questionnaire, which could be considered a limitation.

## Conclusion

GPs and PTs refer less than 25% of patients presenting with LHGP to orthopedic care. GPs and PTs largely adhere to clinical recommendations for assessment regarding the use of history, clinical tests and imaging, but not regarding patient-reported outcome measures. Treatment strategies included mainly pain medication (GPs), exercise and manual therapy (PTs), and advice on physical activity, which is in accordance to recommendations. A large percentage of GPs prescribed opioids as part of pain management, which is not recommended and needs further evaluation. PT intervention duration was generally shorter than recommended. Implementation of evidence-based assessment and treatment provided by GPs and PTs in primary care may improve the care pathway for people with LHGP, from initial contact with health care, through diagnosis and treatment, and the potential referral to orthopedic care.

## Electronic supplementary material

Below is the link to the electronic supplementary material.

Supplementary Material 1

Supplementary Material 2

## Data Availability

The datasets used and/or analyzed during the current study are available from the corresponding author on reasonable request.

## Abbreviations

FABER: Flexion Abduction External Rotation test
FADIR: Flexion Adduction Internal Rotation test
FAIS: Femoroacetabular Impingement Syndrome
GP: General Practitioner
LHGP: Longstanding Hip and Groin Pain
NRS: Numerical Rating Scale
NSAID: Non-Steroidal Anti-Inflammatory Drugs
PROMs: Patient-Reported Outcome Measures
PT: Physical Therapist
ROM: Range Of Motion
VAS: Visual Analog Scale
