# Supplementary material for: Clinical assessment and treatment of patients presenting with longstanding hip and groin pain in primary care: a survey study among physical therapists and general practitioners in Sweden
Source: BMC Musculoskelet Disord. 2025 Mar 3;26:218. doi: 10.1186/s12891-025-08466-6 (PMC11877932; doi:10.1186/s12891-025-08466-6)
Supplement: Supplementary file 1 — Supplementary Material 1 [file 12891_2025_8466_MOESM1_ESM.docx]

# Additional file 1. Perceived importance and ranking

| Perceived importance and ranking of assessment variables | | | | | | |  |  |
| --- | --- | --- | --- | --- | --- | --- | --- | --- |
|  |  | Not at all important, n (%) | Slightly important, n (%) | Somewhat important, n (%) | Very important, n (%) | Extremely important, n (%) |  | Rank |
|  |  |  |  |  |  |  |  |  |
| Diagnosis |  |  |  |  |  |  |  |  |
| Patient History | GP | 0 (0) | 1 (2) | 2 (3) | 17 (27) | 42 (68) |  | 1 |
|  | PT | 0 (0) | 0 (0) | 2 (2) | 14 (14) | 88 (85) |  | 1 |
| Hip ROM | GP | 1 (2) | 2 (3) | 6 (10) | 38 (61) | 15 (24) |  | 2 |
|  | PT | 0 (0) | 2 (2) | 4 (4) | 33 (32) | 65 (63) |  | 2 |
| Isometric Pain Provocation | GP | 6 (10) | 11 (18) | 21 (34) | 21 (34) | 3 (5) |  | 6 |
|  | PT | 1 (1) | 9 (9) | 22 (21) | 43 (41) | 29 (28) |  | 4 |
| Palpation | GP | 1 (2) | 4 (7) | 23 (37) | 27 (44) | 7 (11) |  | 3 |
|  | PT | 4 (4) | 18 (17) | 36 (35) | 30 (29) | 16 (15) |  | 5 |
| Specific Tests | GP | 5 (8) | 15 (24) | 27 (44) | 11 (18) | 4 (7) |  | 4 |
|  | PT | 3 (3) | 13 (13) | 32 (31) | 42 (40) | 14 (14) |  | 3 |
| Imaging Methods | GP | 2 (3) | 14 (23) | 26 (42) | 20 (32) | 0 (0) |  | 5 |
|  | PT | 2 (2) | 59 (57) | 34 (33) | 7 (7) | 2 (2) |  | 6 |
| Assessment of impairments |  |  |  |  |  |  |  |  |
| Tests of physical function | GP | 3 (5) | 8 (13) | 29 (47) | 19 (31) | 3 (5) |  | 1 |
|  | PT | 0 (0) | 1 (1) | 10 (10) | 44 (42) | 49 (47) |  | 1 |
| Manual muscle tests | GP | 7 (11) | 21 (34) | 25 (40) | 9 (15) | 0 (0) |  | 2 |
|  | PT | 3 (3) | 19 (18) | 36 (35) | 40 (39) | 6 (6) |  | 3 |
| Muscle tightness | GP | 6 (10) | 21 (34) | 27 (44) | 8 (13) | 0 (0) |  | 3 |
|  | PT | 9 (9) | 25 (24) | 42 (40) | 25 (24) | 3 (3) |  | 2 |
| Translatory movement of the hip joint | GP | 15 (24) | 19 (31) | 22 (36) | 5 (8) | 1 (2) |  | 4 |
|  | PT | 34 (33) | 39 (38) | 23 (22) | 3 (3) | 5 (5) |  | 4 |
| Dynamometry | GP | 20 (32) | 22 (36) | 17 (27) | 3 (5) | 0 (0) |  | 5 |
|  | PT | 54 (53) | 39 (38) | 9 (9) | 1 (1) | 1 (1) |  | 5 |
| Patient reported outcome measures | GP | 25 (40) | 25 (40) | 11 (18) | 1 (2) | 0 (0) |  | - |
|  | PT | 29 (28) | 46 (44) | 26 (25) | 3 (3) | 0 (0) |  | - |
| Pain scales | GP | 14 (23) | 19 (31) | 19 (31) | 9 (15) | 1 (2) |  | - |
|  | PT | 2 (2) | 22 (21) | 33 (32) | 45 (43) | 2 (2) |  | - |

**Perceived importance of treatment variables**

|  |  | Not at all important, n (%) | Slightly important, n (%) | Somewhat important, n (%) | Very important, n (%) | Extremely important, n (%) |  | Rank |
| --- | --- | --- | --- | --- | --- | --- | --- | --- |
|  |  |  |  |  |  |  |  |  |
| Analgesics Paracetamol | GP | 0 (0) | 6 (10) | 17 (27) | 31 (50) | 8 (13) |  | - |
|  | PT | 5 (5) | 25 (24) | 48 (46) | 20 (19) | 6 (6) |  | - |
| Opioids | GP | 9 (15) | 36 (58) | 14 (23) | 3 (5) | 0 (0) |  | - |
|  | PT | 72 (69) | 26 (25) | 5 (5) | 1 (1) | 0 (0) |  | - |
| Tramadol | GP | 54 (87) | 7 (11) | 1 (2) | 0 (0) | 0 (0) |  | - |
|  | PT | 79 (76) | 21 (20) | 4 (4) | 0 (0) | 0 (0) |  | - |
| NSAIDs Oral | GP | 0 (0) | 6 (10) | 24 (39) | 24 (39) | 8 (13) |  | - |
|  | PT | 5 (5) | 26 (25) | 46 (44) | 24 (23) | 3 (3) |  | - |
| NSAID topical | GP | 32 (52) | 19 (31) | 8 (13) | 3 (5) | 0 (0) |  | - |
|  | PT | 50 (48) | 32 (30) | 20 (19) | 1 (1) | 1 (1) |  | - |
| Physical therapist-led treatment |  |  |  |  |  |  |  |  |
|  | |  |  |  |  |  |  |  |
| Physical activity | GP | 0 (0) | 0 (0) | 4 (7) | 23 (37) | 35 (57) |  |  |
|  | PT | 0 (0) | 1 (1) | 2 (2) | 27 (26) | 74 (71) |  | 2 (59) |
| Exercise therapy | GP | 0 (0) | 0 (0) | 6 (10) | 28 (45) | 28 (45) |  | 2 (58) |
|  | PT | 0 (0) | 0 (0) | 1 (1) | 23 (22) | 80 (77) |  | 1 (44) |
| Passive treatment | GP | 5 (8) | 29 (47) | 19 (31) | 8 (13) | 1 (2) |  | 3 (82) |
|  | PT | 21 (20) | 65 (63) | 16 (15) | 2 (2) | 0 (0) |  | 3 (68) |
| Patient education |  |  |  |  |  |  |  |  |
| Anatomy | GP | 1 (2) | 19 (31) | 28 (45) | 13 (21) | 1 (2) |  | - |
|  | PT | 3 (3) | 5 (5) | 26 (25) | 46 (44) | 24 (23) |  | - |
| Prognosis | GP | 1 (2) | 0 (0) | 12 (19) | 42 (68) | 7 (11) |  | - |
|  | PT | 0 (0) | 2 (2) | 24 (23) | 46 (44) | 32 (31) |  | - |
| Pathophysiology | GP | 1 (2) | 17 (27) | 22 (35) | 19 (31) | 3 (5) |  | - |
|  | PT | 4 (4) | 19 (18) | 28 (27) | 37 (36) | 16 (15) |  | - |
| Pain/Worries | GP | 0 (0) | 0 (0) | 7 (11) | 36 (58) | 19 (31) |  | - |
|  | PT | 0 (0) | 1 (1) | 11 (11) | 48 (46) | 44 (42) |  | - |
| Treatment options | GP | 0 (0) | 1 (2) | 13 (21) | 35 (56) | 13 (21) |  | - |
|  | PT | 2 (2) | 10 (10) | 24 (23) | 52 (50) | 16 (15) |  | - |
| Gp = General Practitioner, PT= Physical therapist. | | | | | | | | |
