# Supplementary material for: Clinical assessment and treatment of patients presenting with longstanding hip and groin pain in primary care: a survey study among physical therapists and general practitioners in Sweden
Source: BMC Musculoskelet Disord. 2025 Mar 3;26:218. doi: 10.1186/s12891-025-08466-6 (PMC11877932; doi:10.1186/s12891-025-08466-6)
Supplement: Supplementary file 2 — Supplementary Material 2 [file 12891_2025_8466_MOESM2_ESM.docx]

# Additional file 2. Survey questions and translation

|  | **Questions** | **Answers** |
| --- | --- | --- |
| Q1. | Vilken är din profession? | 1. Läkare 2. Fysioterapeut |
|  | What is your profession? | 1. General practitioner 2. Physical therapist |
|  |  |  |
| Q2. | Inom vilken sektor arbetar du? | 1. Privat sektor 2. Offenlig sektor 3. Både privat och offentlig sektor |
|  | Within which health care sector do you work? | 1. Private sector 2. Public sector 3. Both private and public sector |
|  |  |  |
| Q3. | Ungefär hur många år har du arbetat inom ditt nuvarande yrke (läkare/fysioterapeut)? | 1. <2 år 2. 2-5 år 3. 6-10 år 4. 11-20 år 5. >20 år |
|  | How many years have you been working within your profession (General practitioner/physical therapist)? | 1. <2 years 2. 2-5 years 3. 6-10 years 4. 11-20 years 5. >20 years |
|  |  |  |
| Q4. | Hur ofta undersöker/behandlar du patienter med långvarig höft- och ljumsksmärta? | 1. Varje vecka 2. Några gånger i månaden 3. Några gånger i halvåret 4. Någon gång per år 5. En gång per år eller mer sällan. |
|  | How often du you assess and/or treat patients with longstanding hip and groin pain? | 1. Every week 2. A few times per month 3. A few times per every 6 months 4. A few times per year 5. Once per year or less |
|  |  |  |
| Q5. | Använder du dig av något/några av följande metoder vid undersökning av patienter med långvarig höft- och ljumsksmärta? 1. Specifika tester (impingement test som t ex FADIR/FABER) 2. Rörlighet i höftled 3. Isometrisk smärtprovokation (t ex statisk höftadduktion) 4. Palpation av t ex muskelfästen och ligament 5. Bilddiagnostik (t ex slätröntgen, MR eller ultraljud) 6. Annat (fritext-ruta) | 1. Ja 2. Nej |
|  | Do you use any of the following methods in assement of people with longstanding hip and groin pain?1. Specific tests (impingement tests such as FADIR/FABER) 2. Hip range of motion 3. Isometric pain provocation (eg static hip adduction) 4. Palpation of muscle attachments and ligaments 5. Imaging (eg x-ray, MRI or ultrasound) 6. Other (free form text) | 1. Yes 2. No |
|  |  |  |
| Q6. | Hur viktiga tycker du följande tester är för diagnostik av långvarig höft- och ljumsksmärta? 1. Specifika tester (impingement test som t ex FADIR/FABER) 2. Rörlighet i höftled 3. Isometrisk smärtprovokation (t ex statisk höftadduktion) 4. Palpation av t ex muskelfästen och ligament 5. Bilddiagnostik (t ex slätröntgen, MR eller ultraljud) | 1. Inte alls viktiga 2. Lite viktiga 3. Ganska viktiga 4. Mycket viktiga 5. Extremt viktiga |
|  | How important do you consider the following tests for the diagnosis of longstanding hip and groin pain? 1. Specific tests (impingement tests such as FADIR/FABER) 2. Hip range of motion 3. Isometric pain provocation (eg static hip adduction) 4. Palpation of muscle attachments and ligaments 5. Imaging (eg x-ray, MRI or ultrasound) | 1. Not important at all 2. Slightly important 3. Somewhat important 4. Very important 5. Extremely important |
|  |  |  |
| Q7. | Rangordna följande tester relaterat till hur viktiga du tycker de är för diagnostik av långvarig höft- och ljumsksmärta där 1=viktigast och 6=minst viktigt. | Specifika tester (impingement test som t ex FADIR/FABER), Rörlighet i höftled, Isometrisk smärtprovokation (t ex statisk höftadduktion), Palpation av t ex muskelfästen och ligament, Bilddiagnosti |
|  | Rank the following tests according to how important you consider them for making a diangosis of longstanding hip and groin pain, where 1=most important and 6= least important | Specific tests (impingement tests such as FADIR/FABER), Hip range of motion, Isometric pain provocation (eg static hip adduction), Palpation of muscle attachments and ligaments, Imaging (eg x-ray, MRI or ultrasound) |
|  |  |  |
| Q8. | Använder du dig av något/några av följande metoder vid undersökning av patienter med långvarig höft- och ljumsksmärta - Funktionstest (t ex balanstest, funktionella rörelser), muskelstramhet, translatorisk rörlighet i höftled | 1. Ja 2. Nej |
|  | Do you use any of the following methods in assessing patients with longstanding hip and groin pain - Functional tests ( eg balance tests, functional movements), muscle tightness assessment, tranlatory movement of the hip joint | 1. Yes 2. No |
|  |  |  |
| Q9. | Använder du någon av följande metoder för att mäta muskelstyrka - Objektiv styrkemätning med dynamometer, Manuella muskeltest | 1. Ja 2. Nej |
|  | Do you use any of the following methods for assessing muscle strength - objective strength assessment using dynamometer, manual muscle tests | 1. Yes 2. No |
|  |  |  |
| Q10. | Hur viktiga tycker du följande tester är för planering av behandling av långvarig höft- och ljumsksmärta - Funktionstest (t ex balanstest, funktionella rörelser), muskelstramhet, translatorisk rörlighet i höftled, Objektiv styrkemätning med dynamometer, Manuella muskeltest | 1. Inte alls viktiga 2. Lite viktiga 3. Ganska viktiga 4. Mycket viktiga 5. Extremt viktiga |
|  | How important do you consider the following tests are for planning treatment of longstanding hip and groin pain - Functional tests ( eg balance tests, functional movements), muscle tightness assessment, tranlatory movement of the hip joint, objective strength assessment using dynamometer, manual muscle tests | 1. Not important at all 2. Slightly important 3. Somewhat important 4. Very important 5. Extremely important |
|  |  |  |
| Q11. | Rangordna följande tester relaterat till hur viktiga du tycker de är för planering av behandling av långvarig höft- och ljumsksmärta från 1=viktigast och 5=minst viktig: | Funktionstest (t ex balanstest, funktionella rörelser), muskelstramhet, translatorisk rörlighet i höftled, Objektiv styrkemätning med dynamometer, Manuella muskeltest |
|  | Rank the following assessments according to how important you consider them for planning treatment of longstanding hip and groin pain, where 1= most important and 6= least important | Functional tests ( eg balance tests, functional movements), muscle tightness assessment, tranlatory movement of the hip joint, objective strength assessment using dynamometer, manual muscle tests |
|  |  |  |
| Q12. | Använder du dig av något/några av följande frågeformulär vid undersökning av patienter med långvarig höft- och ljumsksmärta - Frågeformulär för smärta (t ex VAS eller NRS), frågeformulär för HAGOS, iHOT-33 | 1. Ja 2. Nej |
|  | Do you use any of the following questionnaires when assessing patients with longstanding hip and groin pain - Questionnaires for pain (eg VAS or NRS), questionnaries for hip pain (eg HAGOS, iHOT-33) | 1. Yes 2. No |
|  |  |  |
| Q13. | Hur viktiga tycker du följande frågeformulär är för diagnostik av långvarig höft- och ljumsksmärta - Frågeformulär för smärta (t ex VAS eller NRS), frågeformulär för HAGOS, iHOT-33 | 1. Inte alls viktiga 2. Lite viktiga 3. Ganska viktiga 4. Mycket viktiga 5. Extremt viktiga |
|  | How important do you consider the following questionnaires for diagnosing longstanding hip and groin pain - Questionnaires for pain (eg VAS or NRS), questionnaries for hip pain (eg HAGOS, iHOT-33) | 1. Not important at all 2. Slightly important 3. Somewhat important 4. Very important 5. Extremely important |
|  |  |  |
| Q14. | Rekommenderar/förskriver du något/några av följande smärtlindrande läkemedel till dina patienter - paracetamol, opioider, tramadol, NSAID (tablett), NSAID (salva) | 1. Ja 2. Nej |
|  | Do you recommend and/or prescribe any of the following pain medication to your patients - paracetamol, opioids, tramadol, NSAID (orally), NSAID (topical) | 1. Yes 2. No |
|  |  |  |
| Q15. | Hur viktiga tycker du följande läkemedel är för behandling av patienter med långvarig höft- och ljumsksmärta - paracetamol, opioider, tramadol, NSAID (tablett), NSAID (salva) | 1. Inte alls viktiga 2. Lite viktiga 3. Ganska viktiga 4. Mycket viktiga 5. Extremt viktiga |
|  | How important do you consider the following medications for treating patients with longstanding hip and groin pain - paracetamol, opioids, tramadol, NSAID (orally), NSAID (topical) | 1. Not important at all 2. Slightly important 3. Somewhat important 4. Very important 5. Extremely important |
|  |  |  |
| Q16. | Rekommenderar du någon typ av träning till dina patienter? | 1. Ja 2. Nej |
|  | Do you recommend any kind of exercise to your patients? | 1. Yes 2. No |
|  |  |  |
| Q17. | Om du svarade JA på fråga 16. Vilken/vilka av följande träningsformer rekommenderar du till dina patienter? Du kan fylla i flera alternativ. | 1. Rörlighetsträning 2. styrketräning 3. stabiliseringsträning/neuromuskulär träning 4. plyometrisk träning |
|  | If you answered YES to question 16. Which of the following forms of exercise do you recommend to your patients? You may check several boxes. | 1. Range of motion exercises 2. strength exercises 3. stability/neuromuscular exercises 4. plyometric exercises |
|  |  |  |
| Q18. | Rekommenderar du någon form av fysisk aktivitet till dina patienter? | 1. Ja 2. Nej |
|  | Do you recommend any type of physical activity to your patients? | 1. Yes 2. No |
|  |  |  |
| Q19. | Om du svarade JA på fråga 18 vilken/vilka av följande fysisk aktivitet rekommenderar du till dina patienter? Du kan fylla i flera alternativ. | 1. Vardagsmotion (t ex promenad, trädgårdsarbete) 2. Motionsträning (t ex cykling, löpning, motionsgymnastik) 3. Idrott ( t ex bollsporter, racketsporter, friidrott) |
|  | If you answered YES to question 18. Which of the following forms of physical activity do you recommend to your patients? You may check several boxes. | 1. Everday exercise 2. Endurance exercise 3. Sports |
|  |  |  |
| Q20. | Rekommenderar du några passiva behandlingar till dina patienter? | 1. Ja 2. Nej |
|  | Do you recommend any passive treatments to your patients? | 1. Yes 2. No |
|  |  |  |
| Q21. | Om du svarade JA på fråga 20. Vilken/vilka av följande passiva behandlingar ordinerar du till dina patienter? Du kan fylla i flera alternativ. | 1. Akupunktur/TENS 2. Tejpning/bälte 3. Mjukdelsmobilisering 4. Ledmobilisering |
|  | If you answered YES to question 20. Which of the following forms of passive treatments do you recommend to your patients? You may check several boxes. | 1. Acupuncture/TENS 2. Taping/belts 3. Soft-tissue mobilization 4. Joint mobilization |
|  |  |  |
| Q22. | Hur viktigt tycker du att följande behandlingsalternativ är för behandlingen av långvarig höft- och ljumsksmärta - träning, fysisk aktivitet, passiva behandlingar | 1. Inte alls viktiga 2. Lite viktiga 3. Ganska viktiga 4. Mycket viktiga 5. Extremt viktiga |
|  | How important do you consider the follwong treatment options are for the treatment of longstanding hip and groin pain - Exercise, physical activity, passive treatments | 1. Not important at all 2. Slightly important 3. Somewhat important 4. Very important 5. Extremely important |
|  |  |  |
| Q23. | Rangordna följande behandlingsformer efter hur viktiga du tycker de är för behandlingen av patienter med långvarig höft- och ljumsksmärta där 1=viktigast och 3=minst viktigt: | Träning, fysisk aktivitet, passiva behandlingar |
|  | Rank the following treatments according to how important you consider them for treatment of longstanding hip and groin pain, where 1= most important and 6= least important | Exercise, physical activity, passive treatments |
|  |  |  |
| Q24. | Ger du dina patienter med långvarig höft- och ljumsksmärta någon/några av följande Information/utbildning - Genomgång av anatomiska strukturer, patofysiologisk etiologi, prognos, smärta/oro, behandlingsalternativ | 1. Ja 2. Nej |
|  | Do you provide your patients with longstanding hip and groin pain any of the following information/education - explanation of anatomical structures, pathophysiological aetiologies, prognosis, pain/worries, treatment alternatives | 1. Yes 2. No |
|  |  |  |
| Q25. | Hur viktigt tycker du det är att ge följande information/utbildning till patienter med långvarig höft- och ljumsksmärta Genomgång av anatomiska strukturer, patofysiologisk etiologi, prognos, smärta/oro, behandlingsalternativ | 1. Inte alls viktiga 2. Lite viktiga 3. Ganska viktiga 4. Mycket viktiga 5. Extremt viktiga |
|  | How important do you think it is to provide patients with longstanding hip and groin pain with the following information/education - explanation of anatomical structures, pathophysiological aetiologies, prognosis, pain/worries, treatment alternatives | 1. Not important at all 2. Slightly important 3. Somewhat important 4. Very important 5. Extremely important |
|  |  |  |
| Q26. | Hur stor andel patienter med långvarig höft- och ljumsksmärta uppskattar du att du remitterar till specialistvård? | 1. <25% 2. 25-50% 3. 51-75% 4. >75% |
|  | What percentage of patients with longstanding hip and groin pain do you estimate to referr to tertiary care? | 1. <25% 2. 25-50% 3. 51-75% 4. >75% |
|  |  |  |
| Q27. | Fråga 27 besvaras **ENDAST** av fysioterapeuter. Hur länge i genomsnitt behandlar du en patient med långvarig höft- och ljumsksmärta? | 1. <6 veckor 2. 6 veckor till 3 månader 3. 3-6 månader 4. >6 månader |
|  | Question 27 is answered by physical therapists only. Hur long is your average treatment duration for patients with longstanding hip and groin pain? | 1. <6 weeks 2. 6 weeks to 3 months 3. 3-6 months 4. >6 months |
